# Determinants of low birth weight among newborns delivered at Mettu Karl comprehensive specialized hospital, southwest Ethiopia: a case–control study

**DOI:** 10.1038/s41598-024-54248-w

**Published:** 2024-02-22

**Authors:** Samuel Ejeta Chibsa, Mustafa Adem Hussen, Kenbon Bayisa, Bilisumamulifna Tefera Kefeni

**Affiliations:** 1 Midwifery Department, College of Health Science, Mattu University, Mettu, Ethiopia; 2 Public Health Department, College of Health Science, Mattu University, Mettu, Ethiopia

**Keywords:** Newborn, Low birth weight, Mettu, Ethiopia, Health care, Medical research

## Abstract

Low birth weight is a newborn delivered with birth weight of less than 2500 g regardless of gestational age is called. It is a significant issue affecting over 30 million infants worldwide. Thus, the study determine factors associated with low birth weight among newborns delivered at Mettu Karl Comprehensive Specialized Hospital, Southwest Ethiopia. A facility-based case–control study was conducted with 336 newborns (112 cases and 224 controls) from September 12 to December 23, 2022. The study population was newborns with birth weights of 2500 g to 4000 g as controls and newborns with birth weights < 2500 g were cases. Simple random sampling techniques were used to recruit study participants with a ratio of 1 to 3 cases to controls, respectively. Data was collected by interviews and a checklist. Data were entered and analysed using SPSS version 23. Binary and multivariate logistic regression analyses were computed to identify factors associated with low birth weight, a *p*-value less than 0.05 was used to declare the strength of statistical significance. A total of 327 newborns were contacted, yielding a 97% response rate. MUAC < 23 cm (AOR = 2.72, 95% CI 1.24 to 6.19), inadequate diet diversification (AOR = 4.19, 95% CI 2.04 to 8.60), lack of iron and folic acid supplementation (AOR = 2.94, 95% CI 1.25 to 6.88), history of hypertension (AOR = 2.55, 95% CI 1.09 to 6.00), and lack of nutritional counselling (AOR = 4.63, 95% CI 2.22 to 9.64) were determinants of low birth weight. Low birth weight is linked to residence, maternal MUAC, hypertension history, and ANC visit. Lifestyle modifications, early detection, management, and nutrition information can reduce risk.

## Introduction

Low birth weight (LBW) is a baby delivered with a birth weight of less than 2500 g (5.5 pounds), regardless of gestational age^1^. A large group of infants are born preterm, intrauterine growth-restricted, or both preterm and intrauterine growth-restricted^1,2^. Birth weight is a predictor of perinatal and infant survival, morbidity and mortality, and later risk for developmental disabilities in their lives^3,4^

Globally, more than 30 million newborns are delivered annually; of this, almost one-quarter of them have low birth weight ^1^. The majority of births occur in south-central Asia, with one-third of them weighing less than 2500 g^4,5^. Low birth weight: in Sub-Saharan Africa, 15 percent of them grow up as stunted children, developing different complicated infections that require later hospital admission^3,6^.

Low birth weight is primarily determined by maternal health condition and nutrition status in developing countries, unlike in developed countries, where usually cigarette smoking during pregnancy is the primary cause of low birth weight^7^. Additionally, genetic, socio-demographic, maternal medical illness, intrauterine fetal complications, and environmental factors are predictors of low birth weight across the world^6,8^. In Ethiopia, low birth weights ranged from (10.4 to 17.3%) and this makes Ethiopia grouped under five countries that are accountable for half of global neonatal deaths among Sub-Saharan Africa^9–11^.

The World Health Organisation is set to reduce the burden of low birth weight by as little as 30% by 2025 through nutritional policies on getting affordable, accessible, and appropriate health care for preventing and treating low birth weight^1^. Based on WHO reccomendation, the Ethiopian government declares different strategies to reduce low birth weight and neonatal death^12^. The burden of low birth weight still remained high in Ethiopia^9–11^. The available studies were mainly focuses on the prevalence of low birth weight. therefore, these studies account some of the factors linked with LBW such asnutritional-related factors and nutritional assessment in the the study area. Thus, the study was conducted to determine factors associated with low birth weight among newborns delivered at Mettu Karl Comprehensive Specialised Hospital,South West Ethiopia.

## Methods and materials

### Study design, setting, and period

A facility-based, unmatched case–control study was conducted at Mettu Karl Comprehensive Specialised Hospital from September 12 to December 23, 2022. The hospital is located in Mettu town about 600 km from Addis Ababa, the capital city of Ethiopia. The hospital provides service for in patients and out patients, maternal and child health as well as ART services for patients in the ilu abba bor zone, south-western Ethiopia and Gambella regional states, with a catchment population of more than 2.4 million in 2007 G.C.

### Sourse and study population

The cases were a single alive newborns whose birth weight < 2500 g and had not gross congeital anomalies at Mettu Karl Comprehensive Specialised Hospital (MKCSH) between September 12, 2022 and Decemember 23, 2022. Whereas the controlswere a single alive ewborns weight ≥ 2500 g, but ≤ 4000 g of who were delivered in the same health facilities within 24 h of the delivery of cases and had not gross congeital anomalies.

### Sample size determination

The sample size was determined by using double population proportion formula. By the following assumptions a cases to controls ratio of 1:2. Power = 80%, Zβ = 0.84 for 0.05 significance level, Zα = 1.96 OR = Minimal detectable odds ratio is 2, P1 = proportion of exposure among the cases = 40.9% P2 = the proportion exposed in the control = 24.8%^13^

$${\text{n}} = \left( {{\text{Z}}_{{\alpha /{2}}} + {\text{Z}}_{\beta } } \right)^{{2}} *\left( {{\text{p}}_{{1}} \left( {{1} - {\text{p}}_{{1}} } \right) + {\text{p}}_{{2}} \left( {{1} - {\text{p}}_{{2}} } \right)} \right)/\left( {{\text{p}}_{{1}} - {\text{p}}_{{2}} } \right)^{{2}}$$

### Sampling procedures

After additional consideration of the non-response rate of 5%, the total sample size was 336 (112 cases and 224 controls). A simple random sampling techiques were used to select cases. Whereas controls were selected consequatively at the same and same day of delivey with the ratio of 1 to 2 case to controls within 24 h after delivery. Data was collected each time a low birth weight baby had been delivered until the required sample size was attained.

### Operational defiitions

#### Minimum dietary diversity for women (MDD-W)

The amounts of woman consumes from 10 dietary diversity food groups.

#### Adequate dietary diversity for women

If women consumed at least five different food groups during the previous day or night, from ten food groups.

#### Inadequate dietary diversity for women

If women consumed less than five different food groups during the previous day or night, from ten food groups.

#### Antenatal care (ANC) visits

Which was dichotomized as Yes if a woman has at least one Antenatal care visits and No if a woman has no any Antenatal care visits during the last pregnancy.

### Study variables

#### Sociodemographic factors

Maternal age, educational status, family income, place of residence, occupation, and family size.

#### Obstetric factors

ANC visit, Gravidity, Parity, Gestational age, Pregnancy-induced Hypertension, Labor complication, and vaginal bleeding.

#### Maternal nutritional associated factors

Maternal dietary diversity (MDD-women), Maternal Anthropometric measurements, nutritional counseling during pregnancy, IFA, and Micronutrient supplementations.

#### Maternal medical/lifestyle factors

History of chronic diseases like Hypertension, Diabetes Mellitus, Anemia, TB, Malaria, History of Smoking, Chewing chat, and severe physical work during pregnancy.

### Data collection tools and management procedures

The data were collected through interviews, structured questionnaires, and medical record reviews. The newborn's weight was measured using a balanced Seca scale (German) to the nearest 0.01 g within 1 h of birth. To assure the quality of data two days of training was given to data collectors and supervisors. Trainings started before actual data collection regarding how to approach the study subjects, how to use questionnaires, the data collection procedures, the context of specific questions, and the anthropometric measurement procedures. Minimum Dietary Diversity for Women (MDD-W) was measured by the ten questions developed by FAO and FANTA as a proxy indicator to reflect the micronutrient adequacy of women’s diets. The MDD-W indicator is dichotomous, it returns the value Yes or No. The woman achieves minimum dietary diversity, i.e. Yes, if she consumed at least five different food groups during the previous day or night, and No otherwise (1) Grains, white roots and tubers, and plantains, (2) Pulses (beans, peas and lentils), (3) Nuts and seeds, (4) Milk and milk products, (5) Meat, poultry and fish. (6) Eggs, (7) Dark green leafy vegetables, (8) Other vitamin A-rich fruits and vegetables, (9) Other vegetables, (10) Other fruits^14^. Mid-upper arm circumference (MUAC) was also measured using a non-stretchable MUAC tape according to procedures. The questionaries’ was adapted by reviewing different related literature reviews^15–20^.

### Data processing and analysis

Data were checked for completeness, accuracy, and then cleaned, coded, and were entered to Epi data version 7.0 then exported to SPSS version 23.0 for statistical analysis. Descriptive statistics such as frequency,and percentages were used to describe the study subjects. Binary and multivariate logistic regression analayis was computed to identify factors associated with LBW and a p-value less than 0.05 were used to declare the strength of statistical significances.

### Ethical consideration

This research was performed in accordance with the Helsinki Declaration of Principles. A letter of ethical clearance was obtained from the ethics committee of the College of Health Science at Mattu University, with a reference letter of DPH/157/2022.

### Consent to participate

All the study participants were informed about the purpose of the study and their right to refuse participation or terminate their involvement during the study. Written informed consent was obtained from each study participant.. Information was provided to each study participant before signing the informed consent form on the purpose of the study, data handling, and confidentiality of the information.

## Results

### Socio-demographic characteristics of study participants

A total of 327 were contacted (109 cases and 218 controls) agreed to take part in the study, yielding a 97% response rate. Male constituted in the majority 54% and 59.2% of cases and controls respectively in the study. From the study participants, 35 (32.1%) of the case and 11 (5%) of the control of mothers age were under 20 years (Table 1).

**Table 1:** Soco-demographic characteristics of study participants in Mettu Karl comprehensive specialized hospital, southwest Ethiopia, 2022.

Variables	Cases (n = 109)		Controls (n = 218)	
	Frequency	Percentage	Frequency	Percentage
Age of mothers				
< 20 years	35	32.1	11	5
20–34 years	46	42.2	192	88.1
> 34 years	28	25.7	15	6.9
Residence				
Rural	74	67.8	64	29.3
Urban	35	32.2	154	70.7
Marital status				
Married	91	83.5	200	91.7
Single	8	7.3	8	3.6
Divorced	7	6.5	6	2.7
Widowed	3	2.7	4	1.8
Family size				
< 4	77	70.6	151	69.3
≥ 4	32	29.4	67	30.7
Maternal education				
Cannot read and write	13	11.9	15	6.9
Read and write	16	14.7	14	6.4
Elementary	48	44	65	29.8
Secondary and above	32	29.4	124	56.9
The main occupation of mother				
Farmer	4	3.7	7	3.2
Housewife	59	54	123	56.4
Employee	13	12	42	19.2
Merchant	7	6.4	20	9.2
Student	21	19.3	15	6.9
Daily laborer	5	4.6	10	4.6

Case (n), number of lowbirth newborn; controls (n), number of normal birthnewborn.

### Obstetrics, medical, and nutritional related factors of study participants

In this study, majority of study partcipants 73.3% case and 88.9% control have ANC visits, whereas, 26.7% of cases and 11.1% of controls has no ANC visits. Among study participants 49.5% of cases have no IFA supplementation, whereas 87.6% of controls have IFA supplementation. However only 50.5% of cases have IFA supplementation and 12.4% of control has no IFA supplementation (Table 2).

**Table 2:** Obstetrics, medical and nutritional characteristics of study participants in Mettu Karl comprehensive specialized hospital, Southwest Ethiopia.

Variables	Cases		Controls	
	Number	Percent	number	Percent
Gestational age				
< 37 weeks	36	33.02	33	15.13
37–41 weeks	71	65.13	175	80.27
> 41 weeks	2	1.85	10	4.60
ANC visits				
No	29	26.60	24	11.00
Yes	80	73.40	194	89.00
Weighted during pregnancy				
Yes	47	43.12	175	80.27
No	62	56.88	43	19.73
IFA supplemented				
No	54	49.55	27	12.38
Yes	55	50.45	191	87.62
Chat chewing during pregnancy				
Yes	25	22.93	10	4.58
No	84	77.07	208	95.42
MDD-women				
Inadequate	69	63.30	45	20.64
Adequate	40	36.70	173	79.36
Nutritional counseling				
No	89	81.65	100	45.87
Yes	20	18.35	118	54.13
History of hypertension				
Yes	50	45.87	38	17.44
No	59	54.13	180	82.56
MUAC measurement				
< 23 cm	46	42.20	41	18.80
≥ 23 cm	63	57.80	177	81.20
History of anemia				
No	85	77.90	196	89.90
Yes	24	22.10	22	10.09
Micronutrient supplementation				
No	71	65.13	120	55.04
Yes	38	34.87	98	44.96

Case (n), number of lowbirth newborn; controls (n), number of normal birthnewborn.

### Logestics regression analysis

A significant association were identified between low birth weight were living the rural area, lack of nutritional counseling History of Hypertension, Having ANC visits, Inadequate minimum MDD-women **(**Table 3).

**Table 3:** Multivariate logistic regression analysis of determinants of lowbirth weights in Mettu Karl comprehensive specialized hospital, southwest Ethiopia, 2022.

Variables	Cases (N = 109)	Controls (N = 218)	COR (95% CI)	AOR (95% CI)	*P*-value
Place of residence					
Rural	74	64	5.087 (3.096–8.360)	3.204 (1.58–6.65)	0.01*
Urban	35	154	1	1	
IFA supplemented					
NO	54	27	6.94 (4.00–12.04)	2.943 (1.25–6.88)	0.013*
Yes	55	191	1	1	
MDD-women					
Inadequate	69	45	6.63 (3.98–11.035)	4.195 (2.04–8.60)	0.001***
Adequate	40	173	1	1	
Nutritional counseling					
No	89	100	5.251 (3.01–9.13)	4.630 (2.22–9.64)	< 0.001***
Yes	20	118	1	**1**	
History of hypertension					
Yes	50	38	4.014 (2.40–6.713)	2.559 (1.09–6.00)	0.031*
No	59	180	1	1	
MUAC measurement					
< 23 cm	46	41	3.152 (1.89–5.24)	2.72 (1.24–6.19)	0.012*
≥ 23 cm	63	177	1	1	
ANC visits					
Yes	29	24	2.930 (1.60–5.431)	0.116 (0.51–0.26)	< 0.001***
No	80	194	1	1	

COR, Crude odd ratio; CI, Confidece interval; AOR, 3. Adjusted odd ratio; 1, refernace group.

*p-value < 0.05, **p-value < 0.01. ***p-value < 0.001.

## Discussion

This study reveals that rural women have a higher odds of having a low birth weight than urban mothers. This agreed with individuals reside influences their behaviours, income, and, most significantly, their health and nutrition^15^. The findings are comparable with studies undertaken in Bale, Oromia, Ethiopia, Malaysia, and Yemen, which found that rural pregnant women are more likely to have low birth weights than urban pregnant women^13,16,17^. However, a study conducted in the Jimma Zone found that women living in urban areas had a higher risk of having low-birth-weight babies^18^. This disparity could be attributed to differences in study design, health awareness, geographical location, and demographic features of study participants.

Odds of pregnancy-induced hypertension more risk of developing low birth weight than women who do not have a history of pregnancy-induced hypertension. In fact, high blood pressure may reduce blood flow to the placenta, and as a result, the fetus may not get enough of the nutrients and oxygen needed to grow^15^. Our findings, consistent with studies conducted in north Shewa, Addis Ababa, and Malaysia, showed that pregnant mothers who have a history of pregnancy-induced hypertension were at higher risk of developing low birth weight than pregnant women who have no history of hypertension^9,18,21^ respectively. The possible consistency of the finding could be that hypertension in pregnancy causes narrowing placental blood vessels, will be complicated by utero placental insufficiency, and increases the risk of low birth weight outcomes^9^. However, a study conducted in China showed that hypertensive mothers did not show an increased risk for lowbirth weight^16^. This discrepancy may be due to differences in the study setting, methods, and sociodemographics of the subjects. Additionally, women who were not counselled about nutritional intake during pregnancy had higher odds of having a low birth weight than pregnant women who had been counselled about nutritional intake. Our finding are consistent with a study conducted in North Shewa, Dessie town, Dire Dawa, and Hawassa Ethiopia^10,11,21,22^ respectively. The possible consistency of nutritional counselling may improve their intake habits, and having information about eating a healthy diet can reduce the chance of fetal growth restriction and high blood pressure, reducing the risk of low birth weight in pregnant women^15^.

Iron and folic acid supplementation showed significant assocaiation in this study. Pregnant women who do not receive iron and folic acid supplements have a higher odds of developing low birth weight than their counterparts. Our finding agreed with a study in Nekemte town and north Shewa, Ethiopia^21,23^. This is supported by evidence that IFA supplementation protects against low birth weight as a multiple micronutrient supplement^19^. Additionally, having IFA supplementation during pregnancy decreases the odds of developing a low birth weight compared to the uterine parts of pregnant women^9,20,24^. The possible outcomes could be additional intakes of supplementation, reducing morbidities and risks of congenital malformation.

This study discovered that mothers whose MUAC is less than 23 cm have a higher risk of developing a low birth weight than mothers whose MUAC is greater than or equal to 23 cm. This is supported by a study conducted in the Sidama Zone in south Ethiopia, Yemen, and India^3,17,25^ respectively. This consistency was supported by the available evidence. Low birth weight is a result of undernutrition and the health status of the mother during her pregnancy; MUAC less than normal affects birth weight outcomes^15^.

Inadequate minimum women's dietary diversification increases the risk of being exposed to low birth weight compared to those who had adequate minimum women's dietary diversification during pregnancy. Our findings aligned with a study conducted in Ghana, systematic review reports where women's dietary diversity scores and dietary patterns were found to be protective against low birth weight^26,27^. This consistency could be due to similarities in background characteristics, study design, and study population.

Lastly, women who have antenatal care are 89 percent more protected from low birth than mothers who had non-ANC visits during their recent pregnancy. This is also consistent with studies in Sidama, Bale, Gondar Shewa, Ethiopia, and Italy^3,13,28,29^ respectively. This could be due to the fact that the World Health Organisation strongly recommends during pregnancy the utilisation of antenatal care used for early identification of risky pregnancy and screening for pregnancy complications, as well as early treatments that improve the birth outcomes of pregnancy.

### Strength and limitation of the study

The data was collected with consistent inclusion and exclusion criteria from cases and controls by case control study design to minimise selection bias. Self-reporting methods may lead to recall bias and subjective diagnosis, which may affect the generaliability of findings but not the quality of the study.

## Conclusions

Low birth weight was significantly associated with residence, maternal MUAC, non-compliance with IFA supplementation, hypertension history, nutiritonal counselling, minimum MDD-women, and ANC visit of the mother. Life style Modification, early detection, and management of maternal hypertension, as well as strengthening nutrition information and counselling during pregnancy, will help minimise the risk of low birth weight.

## Data Availability

Data we used in this manuscript were available on behalf of corresponding authors based on reseanble requests.
